# Urinary Podocyte Loss is Associated With Treatment Response in Patients With Primary Nephrotic Syndrome

**DOI:** 10.1016/j.ekir.2025.11.017

**Published:** 2025-11-17

**Authors:** Bartholomeus T. van den Berge, Jitske Jansen, Quinty Leusink, Sanne Kleuskens, Sharon Bootsman, Anne-Els van de Logt, Jack F.M. Wetzels, Bart Smeets, Rutger J. Maas

**Affiliations:** 1 Department of Nephrology, Radboud Institute for Molecular Life Sciences, Radboudumc, Nijmegen, The Netherlands; 2 Department of Pathology, Radboud Institute for Molecular Life Sciences, Radboudumc, Nijmegen, The Netherlands; 3 Department of Medicine 2 (Nephrology, Rheumatology, Clinical Immunology and Hypertension), Institute for Experimental Medicine and Systems Biology, Uniklinik RWTH Aachen, Aachen, Germany

**Keywords:** primary nephrotic syndrome, treatment outcome, urinary podocytes

## Abstract

**Introduction:**

The disease course of primary nephrotic syndrome (PNS) is highly variable, and is difficult to predict at onset. PNS is characterized by podocyte injury and loss. We hypothesized that measurement of urinary podocyte loss is associated with treatment response in patients with PNS.

**Methods:**

We included 21 controls, and 59 patients with PNS (minimal change disease [MCD], *n* =8; focal segmental glomerulosclerosis [FSGS], *n* = 9; membranous nephropathy [MN]. *n* = 42). MCD and FSGS were considered manifestations of the same disease entity, and analyzed as one group. Patients’ baseline clinical and follow-up data were recorded. Urinary sediments were collected and stained for podocyte-specific markers, and analyzed using fluorescence-activated cell sorting (FACS).

**Results:**

In patients with MCD/FSGS, the respective partial and complete remission rates were 24% and 59% during a median follow-up of 12.9 months, and all patients received immunosuppressive treatment. In patients with MN, the respective partial and complete remission rates were 64% and 19% during a median follow-up of 15.5 month, and the majority of patients received immunosuppressive treatment. Patients with PNS had elevated levels of podocyturia when compared with controls, and repeat measurements revealed that podocyturia follows proteinuria course over time, normalizing following complete proteinuria remission. In treatment-responsive immunosuppresive-treated patients with MCD/FSGS, podocyturia at baseline significantly differentiated between early and late treatment responders at 4 weeks, contrary to proteinuria and serum albumin. In symptomatically treated patients with MN, low levels of podocyturia at baseline were associated with spontaneous remission.

**Conclusion:**

Patients with PNS have increased podocyturia compared with healthy individuals. Quantitative detection of podocyturia may have prognostic relevance in patients with PNS.

## Graphical abstract

**Figure:**
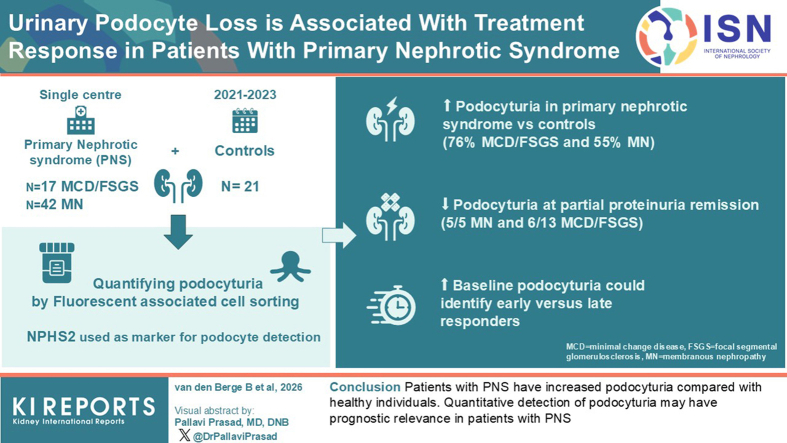

Podocytes are essential for the structure and functioning of the glomerular filtration barrier, and podocyte foot process effacement and detachment are key features of glomerular disease.^1^ Podocytes have a very limited ability to regenerate,^2^^,^^3^ and podocyte loss triggers a cycle of further depletion.^4^ As a result, ongoing podocyte loss can ultimately lead to a critical reduction of the remaining podocyte population. To this end, the podocyte depletion hypothesis states that the outcome of any glomerular injury depends on whether or not the podocyte pool is significantly depleted.^5^ Several studies have shown a relationship between the extent of podocyte depletion and outcome in individuals with glomerular diseases such as Alport syndrome and IgA nephropathy.^6^^,^^7^ In adults with PNS, the most common causes are MCD, FSGS, and MN.^8^ Glomerular injury in PNS is characterized by the absence of inflammation and proliferation. Identification of autoantibodies targeting podocyte-specific proteins, including phospholipase A2 receptor (PLA2R) in patients with MN and nephrin in patients with MCD and FSGS, support the central role of podocytes in PNS pathogenesis.^9^^,^^10^ The disease course in PNS may vary between spontaneous remission and multidrug resistance and progression, and is difficult to predict at onset.^11^, ^12^, ^13^ Measurement of podocyte loss may have added value to assess prognosis and outcome of treatment. Podocyte-specific mRNAs in urine have been proposed as a noninvasive marker for monitoring of podocyte loss. However, mRNA-based methods have high reported variation coefficients, and it is uncertain whether podocyte-specific mRNAs in urine reflect podocyte injury or loss.^14^ In this report, we describe a quantitative method to detect urinary podocytes using FACS. Urinary podocyte loss was measured repeatedly during follow-up in patients with PNS, and compared with the course of proteinuria.

## Methods

### Clinical Characteristics and Outcome Criteria

Adult patients with PNS who were referred to Radboudumc (a university hospital) between 2021 and 2023 were eligible for the study. Inclusion criteria included nephrotic syndrome (proteinuria ≥ 3.5 g/10 mmol or ≥3.5 g/24 h, and hypoalbuminemia), and either histopathological confirmation of PNS or positive serum anti-PLA2R, which was considered diagnostic for MN. Permission for the study was obtained by the local ethical commission for human medical research of the Radboud University Medical Center, Nijmegen, The Netherlands (approval number: 2018-4086); and all participants provided informed consent. All patient data were coded. Urinary samples were obtained at the time of the first outpatient clinic visit, and follow-up visits at our institution. Total protein and creatinine were measured in all samples.

Healthy controls included volunteers and persons who visited our clinic for kidney donor screening. Controls did not have proteinuria and had no history of kidney disease. For definitions of clinical outcomes, we followed the Kidney Disease Improving Global Outcomes 2021 guidelines.^13^ In short, patients were considered in complete remission if proteinuria was ≤ 0.3 g/10 mmol creatinine. Patients were considered in partial remission if proteinuria was reduced to 0.3 g/10 to 3.5 g/10 mmol creatinine, and decreased by > 50% from baseline. Progression was defined as follows: (i) a rise of ≥ 30% in serum creatinine, (2) a rise in serum creatinine > 135 μmol/l, or (iii) other clinical indications for starting immunosuppressive therapy defined by the treating nephrologist. Follow-up data were obtained from referring centers in patients who were not followed-up with at our institution. Estimated glomerular filtration rate at the time of biopsy was calculated using the Chronic Kidney Disease Epidemiology Collaboration equation.^15^ The selectivity index, a measure of glomerular permeability in kidney diseases, was calculated using the excretion ratios of IgG and transferrin. For Extended methods pertaining to definitions of clinical outcomes are presented in the Supplementary Methods.

### FACS for Urinary Podocyte Counting

Freshly collected urine was used for the quantification of urinary podocyte number. Urine was stored at 4 ^◦^C directly after collection and processed within 1.5 hours. In short, urine was centrifuged at 400 rcf for 5 minutes at 4 ^◦^C after which the supernatant was discarded. Cell pellets were washed in FACS buffer (phosphate buffered saline) containing 1% v/v bovine serum albumin) and spun down at 400 rcf for 3 minutes at 4 ^◦^C twice. Cell pellets were diluted in FACS buffer and incubated with primary antibodies for 30 minutes at room temperature. Samples were spun down at 400 rcf for 3 minutes at 4 ^◦^C and washed in FACS buffer twice. Cell pellets were diluted in FACS buffer and incubated with secondary antibodies for 30 minutes in the dark at room temperature. Samples were spun down at 400 rcf for 3 minutes at 4 ^◦^C and washed in FACS buffer twice. During FACS, detection events were gated to select cells and doublet control was performed for both forward and side scatter. Correct gating for cell selection was confirmed by single cell FACS sorting NPHS1^+^ podocytes (derived from induced pluripotent stem cell–derived kidney organoids) as described by Jansen *et al.*^16^ and applying that gating to all patient urine samples. Second antibody controls (cells without primary antibody incubation) were used as a negative control for all patients’ samples individually. Cells were then subsequently counted based on immunofluorescence, using a fluorescence cell sorter (BD Biosciences; FACSAria II SORP 18-color) and DIVA8 software. Information regarding dilutions and primary and secondary antibodies are presented in in Supplementary Table S1.

### Statistical Analysis

Patients with MCD and FSGSs were analyzed as one group, because they are considered part of the same disease spectrum.^10^^,^^17^ All data are expressed as median with interquartile range (IQR), unless stated otherwise. All statistical analyses were performed using IBM SPSS statistics version 29 (IBM) or GraphPad Prism software version 10 (GraphPad Software) and statistical analysis was performed using an unpaired *t* test or, when appropriate, 1-way analysis of variance followed by Dunnett’s *post hoc* test, unless stated otherwise.

## Results

### Baseline Clinical Characteristics and Clinical Outcomes of Study Participants

Baseline clinical characteristics of controls and patients with MCD/FSGS and MN are summarized in Table 1. In summary, 21 controls, 17 patients with MCD/FSGS, and 42 patients with MN were included. All patients had nephrotic syndrome at the time of the first urine sampling. In total, 34 patients were included at the time of disease onset, 11 presented at the time of referral because of persistent nephrotic syndrome after a period of initial symptomatic treatment (MN: *n* = 8), or treatment-resistant nephrotic syndrome (MCD/FSGS: *n* = 3), and 14 patients were included at the time of a relapse. No statistically significant differences were observed in clinical characteristics at baseline or treatment outcome between patients included at first onset, persistent nephrotic syndrome or relapse, for both patients with MCD/FSGS and those with MN. Clinical outcome measures of patients with MCD/FSGS and MN are summarized in Table 2. Median follow-up in the MCD/FSGS patient cohort was 12.9 months, and all patients received immunosuppressive treatment, which mostly consisted of high-dose prednisolone. Overall, 82% (14/17) reached remissions. No statistically significant differences were observed in treatment modality, treatment duration, or treatment outcome between MCD and FSGS patients. In the MN patient cohort median follow-up time was 15.5 months. Immunosuppressive treatment was initially withheld in 24 patients (detailed below), and resulted in spontaneous remission in 9 patients. In total, 31 patients received immunosuppressive treatment during follow-up. Overall, 83% (35/42) of patients with MN reached remission during follow-up. In addition, 74% received immunosuppressive treatment, either with rituximab, prednisone, and cyclophosphamide, or a combination (Table 2).

**Table 1:** Baseline clinical characteristics of controls and patients with primary nephrotic syndrome

Characteristics	Controls			MCD/FSGS			Membranous nephropathy		
	*n*	Median	IQR	*n*	Median	IQR	*n*	Median	IQR
Age (yrs)	21	36	23–60	17	54	38–70	42	60	57–68
Sex (% male)	21	48%		17	59%		42	76%	
Ethnicity (% European)	21	100%		17	100%		42	98%	
Serum creatinine (μmol/l)				17	117	88–161	42	94	76–112
eGFR (ml/min per 1.73 m^2^)				17	54	34–77	42	72	57–91
Serum albumin (g/l)				16	18	15–24	42	24	18–29
UPCR (g/10 mmol)	21	0.08	0.07–0.13	17^a^	5.6	4.1–8.7	42^a^	6.0	3.7–9.0
MCD/FSGS				17	8/9				
PLA2R associated MN (yes/no)							42	29/13	
Disease stage at inclusion (first onset/persistent/relapse)				17	12/3/2		42	22/8/12	

eGFR, estimated glomerular filtration rate; IQR, interquartile range; MCD/FSGS, minimal change disease/focal segmental glomerulosclerosis; UPCR, urinary protein-to-creatinine ratio.

Continuous data are expressed as median (IQR). Percentages (%) are given as percentage of total cohort.

a All patients had nephrotic syndrome at time of disease presentation (≥ 3.5 g/10 mmol).

**Table 2:** Clinical outcome measures in patients with primary nephrotic syndrome

Characteristics	MCD/FSGS			Membranous nephropathy		
	*n*	Median	IQR	*n*	Median	IQR
Time to IS-induced partial remission (mohs)	14	0.9	0.7–1.9	26	3.7	2.6–9.6
Time to spontaneous partial remission (mths)				9	17.9	8.2–34.9
(% of total reaching PR/CR/NR)	17	(24%/59%/18%)		42	(64%/19%/17%)	
Time to follow-up (mos)	17	12.9	11.5–17.3	42	15.5	9.4–25.8
Immunosuppression received (%)	17	100%		31	74%	
No immunosuppression received (%)				11	26%	
Monotherapy (%)	13	77%		10	24%	
Prednisone (%)	12	71%				
Tacrolimus (%)	1	6%				
Rituximab (%)				10	24%	
Combination therapy (%)	4	24%		21	50%	
Prednisone + tacrolimus (%)	2	12%				
Prednisone + MMF (%)	2	12%		1	2%	
Prednisone + cyclophosphamide (%)				8	19%	
Prednisone + cyclophosphamide + RTX (%)				10	24%	
Combination of several therapies (%)				2	5%	

CR, complete remission; IQR, interquartile range; IS, immunosuppressive treatment; MCD/FSGS, minimal change disease/focal segmental glomerulosclerosis; MMF, mycophenolate mofetil; MN, membranous nephropathy; NR, non remission; PR, partial remission; RTX, rituximab.

Continuous data are expressed as median (IQR). Percentages (%) are given as percentage of total cohort.

### Increased Podocyturia in Patients With PNS

**Figure 1:**
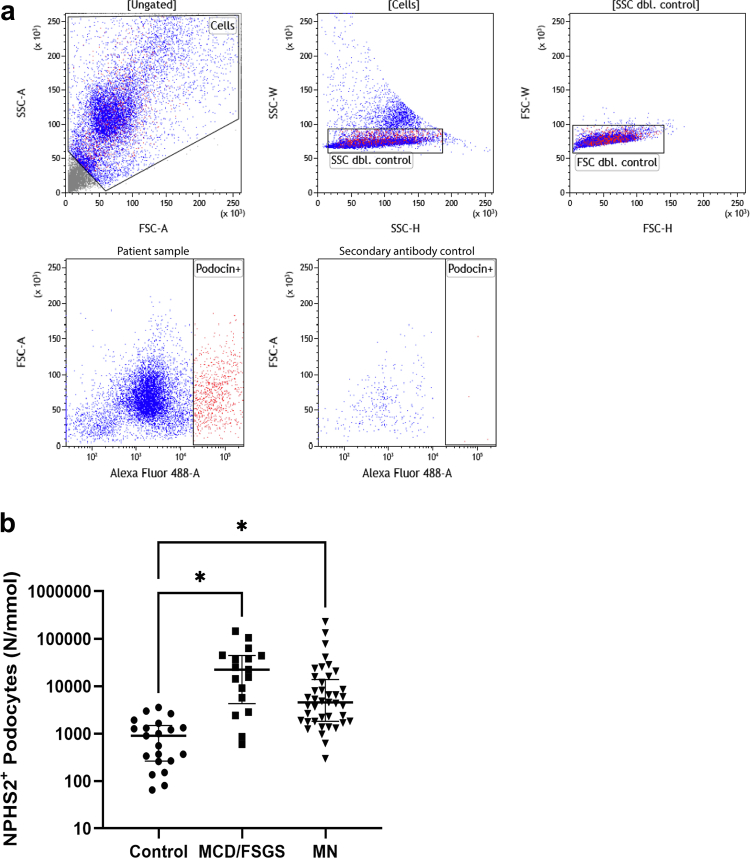
Podocyturia in controls and patients with primary nephrotic syndrome. (a) Representative overview of fluorescence-activated cell sorting gating in patient with primary nephrotic syndrome. First, intact cells were selected (P1). Doublet control in both side scatter (P2) and forward scatter (P3) was applied to allow for singular cell counts. Finally, single cell NPHS2^+^ podocytes (P4) were counted. Gating for P4 was set using a second antibody control to correct for slight changes in (auto)fluorescent background levels between measurements. (b) NPHS2^+^ podocyte loss. NPHS2^+^ podocyte loss was significantly increased in both MCD/FSGS and MN patients when compared with controls. FSC dbl: forward scatter doublet; MCD/FSGS, minimal change disease/focal segmental glomerulosclerosis; MN, membranous nephropathy; NPHS1, nephrin; NPHS2, podocin; SSC dbl, side scatter doublet. ∗*P* ≤ 0.05 *t*-test.

A FACS assay to detect single-cell urinary podocyte loss was set-up, based on the detection of podocin (NPHS2)-positive cells in fresh urine samples (Figure 1a). Initial tests in 36 urine samples of PNS and 10 control samples showed that NPHS2-based detection of urinary podocytes showed better discrimination between patients with PNS and controls compared with NPHS1-based detection of urinary podocytes (Supplementary Figure S1), and NPHS2 was therefore chosen as marker for further analysis. In addition, costaining of NPHS2 and podocalyxin (PODXL) of urinary podocytes in patients with MCD/FSGS (*n* = 8) showed that most urinary podocytes were NPHS2^+^PODXL^−^ (72% ± 11%) or NPHS2^+^PODXL^+^ (24% ± 10%) positive. Only a small percentage of urinary podocytes was NPHS2^−^PODXL^+^ (4% ± 1%) positive, suggesting that NPHS2 is a better marker for total urinary podocyte detection. Median percentage of NPHS2^+^ podocyte loss per total cell number was 4% (IQR: 1%–14%). Using NPHS2 as podocyte marker in all urine samples, there remained significant differences between patients with PNS (59 urine samples) and controls (21 urine samples) (Figure 1b). Using the highest podocyturia in controls of 4000/mmol creatinine as cut-off, 23 of 42 patientswith MN (55%) and 13 of 17 patients with MCD/FSGS (76%) had elevated podocyturia at baseline. As described previously,^18^ we found no statistically significant association between (NPHS2^+^) podocyte loss and chronic kidney disease stage at inclusion in patients with PNS, suggesting that podocyte loss is independent of kidney function in our cohorts (Supplementary Figure S2). Notably, no statistically significant correlation was observed between NPHS2^+^ podocyte loss and age in controls, suggesting that podocyte loss is not affected by age in healthy individuals (Supplementary Figure S3).

### Podocyturia Normalizes Following Complete Proteinuria Remission

**Figure 2:**
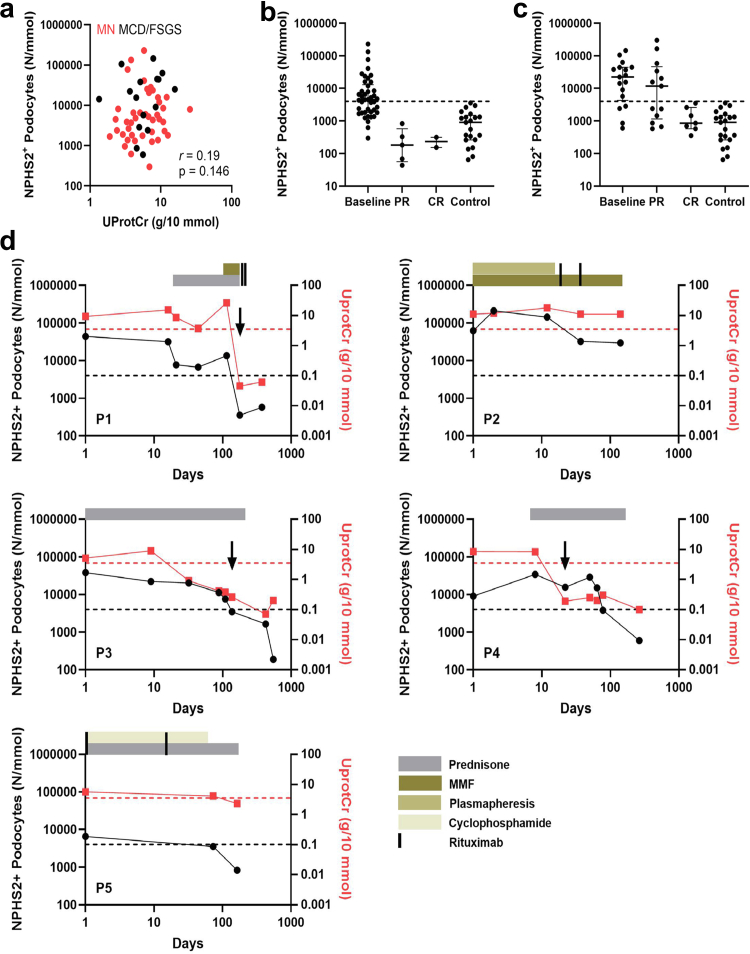
Podocyturia over time in patients with primary nephrotic syndrome. (a) NPHS2^+^ podocyte loss versus proteinuria (UProtCr) in patients with primary nephrotic syndrome at disease onset. No statistically significant correlation was found. (b) NPHS2^+^ podocyturia at baseline and per proteinuria remission status in patients with MN. (c) NPHS2^+^ podocyturia at baseline and per proteinuria remission status in patients with MCD/FSGS. (d) NPHS2^+^ podocyte loss over time in 5 representative patients with primary nephrotic syndrome during immunosuppressive treatment (P1–P5). P1, P3, and P4 had MCD, P2 had treatment-resistant FSGS, and P5 had MN. The black arrows indicate the first measurement after reaching CR (≤ 0.3 g/10 mmol). The red dotted line indicates nephrotic range proteinuria of 3.5 g/10 mmol. The black dotted line indicates the upper limit of control NPHS2^+^ podocyte loss (4000 N/mmol). CR, complete proteinuria remission; FSGS, focal segmental glomerulosclerosis; MCD/FSGS, minimal change disease/focal segmental glomerulosclerosis; MCD, minimal change disease; MMF, mycophenolate mofetil; MN, membranous nephropathy; NPHS2, podocin; PR, partial proteinuria remission; UprotCR, urinary protein creatinine ratio.

Repeat measurement of urinary podocytes was performed in 30 patients with PNS (MN: *n* = 13; MCD/FSGS: *n* = 17). Patients had a median of 1.5 (IQR: 1.0–2.3) follow-up measurements. Overall, there was no statistically significant correlation between podocyturia and urinary protein-to-creatinine ratio (Figure 2a). We observed a reduction of podocyturia to levels comparable with controls at the time of proteinuria remission in patients with PNS (MN: Figure 2b; MCD/FSGS: Figure 2c). A decrease in podocyturia was noted in all patients with MN at the time of partial proteinuria remission (5/5), and in 6 of 13 patients with MCD/FSGS with partial remissions. In patients with MN, 4 of 5 manifested negative serum anti-PLA2R at partial remission, while 2 of 2 had negative anti-PLA2R at complete remission. In some patients, numerous follow-up measurements were obtained during their disease course (Figure 2d (P1–P5)). In these patients, we observed that podocyte loss decreased in parallel with proteinuria in patients who reached remissions, and remained high in patients who did not manifest proteinuria response (P2).

### Association Between Podocyturia and Outcome of Immunosuppresive-Treatment in Patients With MCD/FSGS

**Figure 3:**
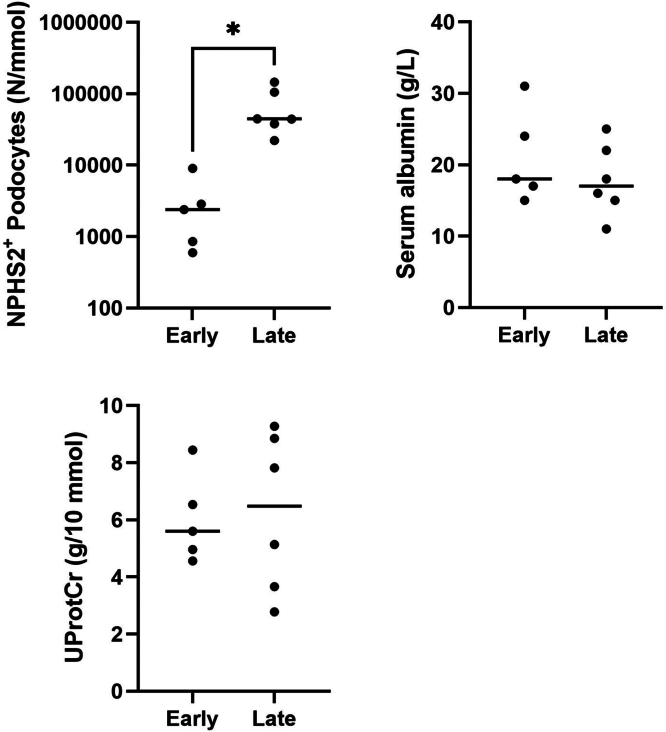
Podocyturia differentiates between early and late responders in patients with minimal change disease/focal segmental glomerulosclerosis. NPHS2^+^ podocyte loss significantly differentiated between early (≤ 4 weeks) and late (> 4 weeks) in patients with treatment-responsive minimal change disease/focal segmental glomerulosclerosis (*n* = 11). The UProtCr (*n* = 11) or serum albumin (*n* = 11) could not differentiate between early or late responders at 4 weeks in patients with steroid-sensitive minimal change disease/focal segmental glomerulosclerosis. NPHS2, podocin; UprotCr, urinary protein-to-creatinine ratio. ∗*P* ≤ 0.05 (*t-*test).

Twelve patients with MCD/FSGS received initial treatment with high-dose prednisolone, and 11 responded with proteinuria remission. Podocyturia at baseline significantly differentiated between early and late treatment responders at 4 weeks, whereas the urinary protein-to-creatinine ratio and serum albumin could not (Figure 3). Median time-to-remission was 0.8 (IQR: 0.5–0.8) months and 2.3 (IQR: 1.2–5.4) months for early and late responders, respectively. There was no statistically significant difference between MCD and FSGS in early (2 vs. 3) or late (4 vs. 2) treatment response, respectively. No statistically significant differences were observed for baseline clinical characteristics, experimental measurements, or treatment outcomes between patients with MCD or FSGS.

### Association Between Podocyturia and Outcome of Symptomatic Treatment in Patients With MN

In patients with MN, urinary high- and low-molecular-weight proteins were measured to assess the probability of proteinuria remission without immunosuppressive treatment.^19^, ^20^, ^21^, ^22^ The sediments of these portions were used to measure podocyturia to assess the prognostic value of podocyturia for therapeutic decisions in patients with MN. We observed weak correlations between podocyturia and urinary proteins, including a statistically significant correlation with urinary beta-2-microglobulin (Table 3). No statistically significant difference was observed in podocyturia between initially symptomatically treated patients with MN and immunosuppressive-treated patients with MN.

**Table 3:** Correlations of NPHS2^+^ podocyte loss with other urinary parameters

Characteristics	Membranous nephropathy		
	*n*	*Rho*	*P*-value
Age (yrs)	42	0.191	0.23
UPCR (g/10 mmol)	42	0.199	0.21
IgG/creatinine (mg/mmol)	40	0.270	0.09
Selectivity index	38	0.278	0.09
Alpha-1-microglobulin/creatinine (mg/mmol)	40	0.137	0.40
Alpha-1-microglobulin/min (μg/min)	40	0.148	0.36
Beta-2-microglobulin/creatinine (mg/mmol)	40	0.328	0.04
Beta-2-microglobulin/min (μg/min)	40	0.288	0.07

UPCR, urinary protein creatinine ratio.

Statistically significant *P*-values are < 0.05.

In Table 4, we present a summary of the baseline characteristics of patients with MN according to the outcome of initial symptomatic treatment and initial immunosuppressive treatment. Patients who reached spontaneous remissions had less severe nephrotic syndrome than those who did not, as indicated by proteinuria and serum albumin, respectively. In patients who received initial symptomatic treatment (*n* = 24), patients with control levels of podocyturia at baseline (≤ 4000 NPHS2^+^ podocytes/mmol) more often reached spontaneous proteinuria remission (62%) than patients with podocyturia above control levels (> 4000 NPHS2^+^ podocytes/mmol) (9%) (χ^2^, *P* < 0.01). No statistically significant differences were observed for baseline clinical characteristics, experimental measurements, or treatment outcomes between PLA2R^+^ or PLA2R^−^ patients with MN.

**Table 4:** Baseline characteristics of MN patients with and without initial immunosuppressive treatment

Characteristics	Without initial IS treatment						*P*-value vs. spon. rem	With initial IS treatment		
	Spon. rem			No spon. rem						
	*N*	Median	IQR	*N*	Median	IQR		*N*	Median	IQR
Age (yrs)	9	57	46–62	15	64	57–70	0.21	18	61	57–69
Serum creatinine (μmol/l)	9	77	63–95	15	94	81–109	0.46	18	102	85–132
eGFR (ml/min per 1.73 m^2^)	9	96	74–104	15	66	58–90	0.53	18	66	49–78
Serum albumin (g/l)	9	27	26–32	15	21	14–26	0.02	18	23	17–29
UPCR (g/10 mmol)	9^a^	3.50	3.00–3.85	15^a^	6.90	3.80–9.50	0.01	18^a^	7.15	5.20–9.20
NPHS2^+^ podocytes/creatinine (N/mmol)	9	1886.4	1315.1–3770.4	15	4815.2	2188.2–27943.1	0.05	18	6111.29	2221.30–15663.48
IgG/creatinine (mg/mmol)	9	18.9	5.5–39.6	14	15.7	8.9–99.2	0.48	17	9.8	4.9–79.1
Selectivity index	8	14.5	8.7–24.1	13	17.6	13.5–29.5	0.21	17	18.8	14.8–28.7
Alpha-1-microglobulin/creatinine (mg/mmol)	9	6.7	2.5–9.5	14	3.4	2.6–11.2	0.98	17	4.0	1.2–5.5
Alpha-1-microglobulin/min (μg/min)	9	49.7	32.5–130.0	14	44.7	30.3–132.4	0.89	17	69.5	40.9–127.9
Beta-2-microglobulin/creatinine (mg/mmol)	9	0.052	0.020–0.609	14	0.085	0.033–0.500	0.46	17	0.062	0.026–0.519
Beta-2-microglobulin/min (μg/min)	9	0.413	0.267–6.036	14	0.947	0.345–4.006	0.67	17	1.721	0.621–4.555

IQR, interquartile range; IS, immunosuppressive; spon. rem, spontaneous remission; UPCR, urinary protein creatinine ratio.

Continuous data are expressed as median (IQR). *t-*test was performed for comparison. Statistically significant *P*-values are < 0.05.

a All patients had nephrotic range proteinuria at time of presentation (≥ 3.5 g/10 mmol).

## Discussion

The findings of this study support the relevance of podocyte loss as a noninvasive biomarker of disease activity and outcome in patients with PNS. We successfully set up a FACS-based method to quantify urinary podocyte loss using NPHS2 as a podocyte-specific marker. The majority of patients with PNS had elevated levels of podocyturia when compared with healthy controls. Repeat measurements during treatment revealed that podocyturia followed proteinuria over time, reaching control levels at the time of complete proteinuria remission. In nephrotic patients with PNS, podocyturia in the range of controls (≤ 4000/mmol creatinine) was associated with favorable long-term outcomes, as demonstrated by the increased likelihood of spontaneous proteinuria remission in patients with MN and early prednisone-induced proteinuria remission in patients with MCD/FSGS.

It has been demonstrated that podocytes can be detected in urine from healthy persons and patients with glomerular diseases including PNS,^23^^,^^24^ and viable podocytes in urine have been used to obtain podocyte-lineage cell lines.^25^ Subsequently, quantitative measurement of podocyte markers in urine has been proposed to evaluate glomerular diseases. For example, detection of urinary nephrin has been proposed as a sensitive marker of preeclampsia and diabetic nephropathy.^26^^,^^27^ Other researchers have performed quantitative measurement of urinary podocyte-specific mRNAs, and showed correlations between relative increases of NPHS2 mRNA excretion and disease severity and progression in patients with MCD/FSGS and Alport syndrome.^7^^,^^14^ The concentrations of urinary podocyte-specific transcripts were close to the detection limit, and quantifications were poorly reproducible (unpublished data). For this reason, and because we aimed to assess the numeric loss of podocytes in urine, we pursued a different method. Detection of urinary podocytes has been performed with specific anti- PODXL or Wilms tumor 1 antibodies.^28^, ^29^, ^30^, ^31^, ^32^ Achenbach *et al.* and others have shown that parietal epithelial cells, which share developmental origins with podocytes, also express PODXL and Wilms tumor 1 and are readily excreted into the urine during active glomerular disease.^33^, ^34^, ^35^ In addition, Hara *et al.*^36^ showed that urinary PODXL may not originate from detached podocytes, but from membrane shedding in response to podocyte injury. Because we aimed to measure podocytes specifically, keeping in mind the podocyte depletion hypothesis, and that none of the previously published articles showed any associations with disease outcome on an individual level, we addressed the limitations of previous studies by using highly podocyte-specific markers NPHS1 and NPHS2, performing a reproducible FACS-based detection with specific gating to identify whole podocytes.

Studies that relied on PODXL as a podocyte marker reported urinary podocyte loss in ranges between 0/d and 400/d.^29^^,^^37^^,^^38^ By contrast, we detected a median of 900 podocytes per mmol creatinine in controls. As an example, this would imply a total daily podocyturia of 10,800 cells in a person who excretes 12 mmol of creatinine that day. Based on biopsy studies, it has been estimated that healthy individuals lose 2.3 podocytes per glomerulus/yr, or 5.63 million podocytes per kidney/yr.^7^^,^^39^ Average nephron numbers are estimated to be approximately 1 million per kidney.^40^ Based on these data, daily podocyte loss would be estimated between 12,000 to 30,000 in healthy individuals, which is in agreement with our findings.

Although several methods to measure podocyte markers in urine have been described, few studies have performed longitudinal sampling, or evaluated the usefulness of initial podocyte markers to predict proteinuria remission. We observed that podocyturia decreased along with proteinuria and reached control levels at the time of complete proteinuria remission. These findings are in line with results in a study that relied on urinary NPHS2 mRNA quantification.^14^ Findings in patients with partial remissions were more variable. Although podocyturia decreased to control levels in all patients with MN at the time of partial remission (*n* = 5), this was only observed in 6 of 13 patients with MCD/FSGS (46%) at the onset of partial remission. The discrepancy may be related to the fact that the proteinuria response tends to be slower in MN compared with MCD/FSGS. Importantly, 4 of 5 patients with MN with partial remissions had a negative serum anti-PLA2R. This suggests that the decrease in podocyturia may coincide with the disappearance of immunological disease activity. Recently, antinephrin autoantibodies have been identified in patients with MCD/FSGS.^10^^,^^41^ We did not have serum available to evaluate antinephrin in our patients with MCD/FSGS.

We acknowledge several limitations of our study. Although we developed a specific method to detect podocytes in urine, the method requires fresh urine and is not suitable for high throughput testing. The sample size of our cohort is limited, including a few patients with treatment-resistant PNS, and the predictive value of podocyturia for a favorable outcome in PNS requires validation. Finally, longitudinal measurement of podocyturia was not performed systematically. Freund *et al.*^42^ have developed a method for the gentle fixation of urine cell pellets enabling delayed processing for flow cytometric analysis, and we recommend that this method be deployed for more systematic measurements in future studies. Despite these limitations, our study suggests that the proposed method may be used to monitor the disease course and predict treatment response in patients with PNS. Further studies are needed to evaluate the relationship between proteinuria, podocyturia, and podocyte-specific autoantibodies in patients with PNS, which may allow improved definitions of disease remission.

## Disclosure

All the authors declared no competing interests.

## Supplementary Material

Supplementary File (PDF) **Supplementary Methods. Figure S1.** Podocyturia in controls and patients with PNS. **Figure S2.** Podocyturia and CKD stages in PNS patients. **Figure S3.** Podocyturia and age in controls.

## Footnotes

Supplementary File (PDF)

**Supplementary Methods.**
**Figure S1.** Podocyturia in controls and patients with PNS. **Figure S2.** Podocyturia and CKD stages in PNS patients. **Figure S3.** Podocyturia and age in controls.
