# Supplementary material for: Urinary Podocyte Loss is Associated With Treatment Response in Patients With Primary Nephrotic Syndrome
Source: Kidney Int Rep. 2025 Nov 17;11(2):103692. doi: 10.1016/j.ekir.2025.11.017 (PMC12800585; doi:10.1016/j.ekir.2025.11.017)
Supplement: Supplementary File (PDF) — Supplementary Methods. Figure S1. Podocyturia in controls and patients with PNS. Figure S2. Podocyturia and CKD stages in PNS patients. Figure S3. Podocyturia and age in controls. [file mmc1.pdf]

Urinary podocyte loss is associated with treatment response in patients with primary nephrotic syndrome

Bartholomeus T van den Berge[1,2], Jitske Jansen[3], Quinty Leusink[1], Sanne Kleuskens[2], Sharon Bootsman[2], Anne-Els van de Logt[1], Jack FM Wetzels[1], Bart Smeets[2], Rutger J Maas[1¥]

[1]Department of Nephrology, Radboud Institute for Molecular Life Sciences, Radboudumc, Nijmegen, The Netherlands. [2]Department of Pathology, Radboud Institute for Molecular Life Sciences, Radboudumc, Nijmegen, The Netherlands. [3]Institute for experimental medicine and systems biology, Uniklinik RWTH Aachen, Aachen, Germany.

[¥]Corresponding author

Rutger.Maas@radboudumc.nl

Geert Grooteplein Zuid 10, 6525 GR Nijmegen, The Netherlands

Supplementary Material

Page 2: Supplementary figure S1. "Podocyturia in controls and patients with PNS."

Page 3: Supplementary figure S2. "Podocyturia and CKD stages in PNS patients."

Page 4: Supplementary figure S3. "Podocyturia and age in controls."

Page 5: Supplementary Methods.

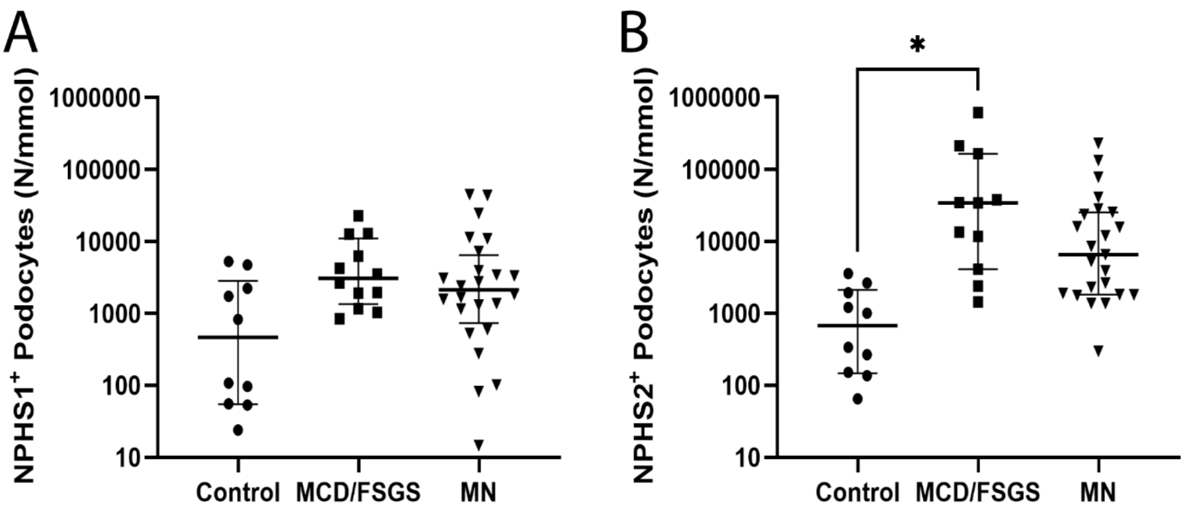

Supplementary figure S1. Podocyturia in controls and patients with PNS. (A) No significant differences were observed in either MCD/FSGS or MN patients when compared to controls for NPHS1$^+$ podocyte loss. (B) NPHS2$^+$ podocyte loss was significantly increased in MCD/FSGS patients when compared to controls. MCD/FSGS: minimal change disease/focal segmental glomerulosclerosis; MN: membranous nephropathy; NPHS1: nephrin; NPHS2: podocin. *: $p \leq 0.05$ (one-way ANOVA).

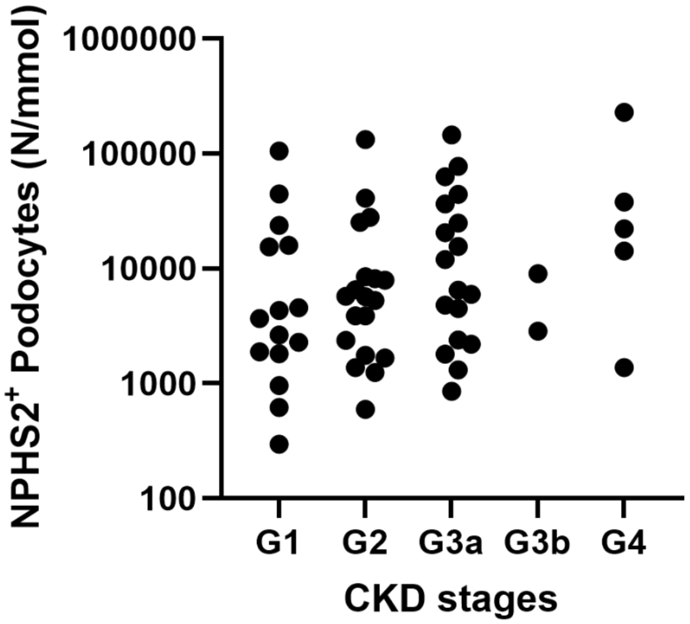

Supplemental figure S2. Podocyturia and CKD stages in PNS patients. NPHS2$^+$ podocyte loss in patients was divided into CKD stages. CKD stages were as follows: G1 (eGFR ≥90 mL/min/1.73 m^2) for normal kidney function, G2 (eGFR 60–89 mL/min/1.73 m^2) for mildly decreased function, G3a (45–59 mL/min/1.73 m^2) for moderately decreased function, G3b (30–44 mL/min/1.73 m^2) for moderately to severely decreased function, G4 (15–29 mL/min/1.73 m^2) for severely decreased function, and G5 (<15 mL/min/1.73 m^2 or on dialysis) for kidney failure. CKD stage G5 (<15 mL/min/1.73 m^2 or on dialysis) was not included since MCD or FSGS patients may suffer from acute kidney failure (N=2 in our cohort), but often still respond well to treatment resulting in recovery of kidney function. In those specific instances, the eGFR at inclusion does not reflect their true kidney function. No statistical significant association was found between NPHS2$^+$ podocyte loss and CKD stage (Kruskal-Wallis test). NPHS2: podocin.

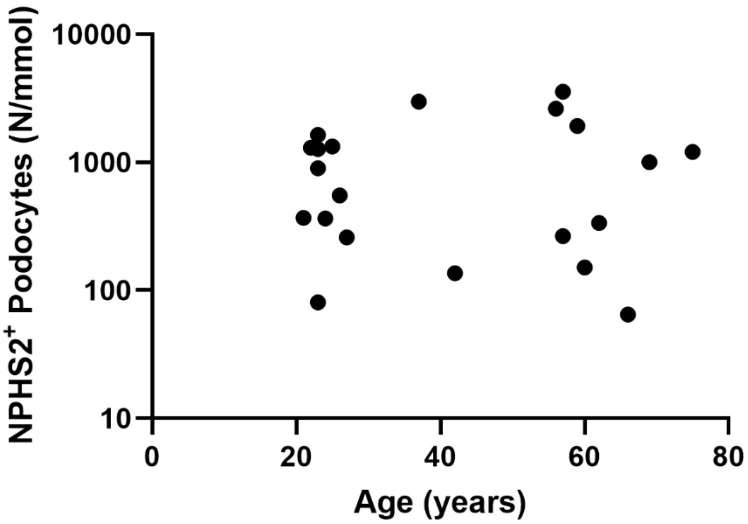

Supplementary figure S3. Podocyturia and age in controls. NPHS2^{+} podocyte loss in controls. No statistical significant correlation was found between NPHS2^{+} podocyte loss and age. NPHS2: podocin.

Supplementary Methods

Clinical outcome definitions

Spontaneous remission is described as obtaining (partial or complete) remission without the use of immunosuppressive treatment. Since spontaneous remission occurs frequently in MN (Hofstra 2011. CJASN; Polanco 2010. JASN), our university medical center routinely employs timed urinary measurements to predict outcome of symptomatic treatment (van den Brand 2011. CJASN).

All relevant PNS patients' clinical data was collected by accessing electronic medical records, which included the pathology report for biopsy-confirmed diagnosis. Collected relevant information included age at time of biopsy, sex, ethnicity, laboratory results including serum creatinine, urinary protein to creatinine ratio, serum albumin, anti-PLA2R, pathologist's report and immunosuppressive treatment regimen received.

Table S1. Immunofluorescent staining overview of used chemicals, antibodies, working dilutions and antigen retrieval buffers.

**Primary antibody or chemical**	**Working dilution**	**Secondary antibody**	**Working dilution**	**Buffer**
Urinary podocyte count				
Anti-Podocin antibody (P0372, Sigma)	1:100	Donkey anti-rabbit Alexa Fluor™ 488 (A21206, Thermo Fisher)	1:200	FACS buffer: PBS containing 1% *v/v* BSA
Human Nephrin Antibody (AF4269, R&D Systems)	1:100	Donkey anti-sheep Alexa Fluor™ 647 (A21448, Thermo Fisher)	1:200	

Table S2. Resource table

**Software and Algorithms**		
Adobe Illustrator CC 2021	Adobe Systems Inc.	RRID:SCR_010279
Adobe Photoshop CC 2021	Adobe Systems Inc.	RRID:SCR_014199
GraphPad Prism version 10	GraphPad Software Inc.	RRID:SCR_002798
SPSS version 29	IBM SPSS statistics 29	RRID:SCR_016479
Kaluza version 2.2	Beckman Coulter	RRID:SCR_016182
